# *catena*-Poly[[bis­(μ-3-amino­pyrazine-2-carboxyl­ato)-κ^3^ *N* ^1^,*O*:*O*;κ^3^ *O*:*N* ^1^,*O*)dilithium]-di-μ-aqua]

**DOI:** 10.1107/S1600536810020647

**Published:** 2010-06-05

**Authors:** Wojciech Starosta, Janusz Leciejewicz

**Affiliations:** a Institute of Nuclear Chemistry and Technology, ul.Dorodna 16, 03-195 Warszawa, Poland

## Abstract

The title compound, [Li(C_5_H_4_N_3_O_2_)(H_2_O)]_*n*_, is composed of centrosymmetric dinuclear units, in which the Li^I^ ions are bridged by two carboxyl­ate O atoms donated by two ligands. The dinuclear unit is nearly planar [r.m.s. deviation = 0.0125 (2) Å]. The Li^I^ ion is coordinated by an *N*,*O*-chelating ligand, a bridging carboxyl­ate O atom from another ligand and two bridging water O atoms in a distorted trigonal-bipyra­midal geometry. The water O atoms bridge the dinuclear units into a polymeric mol­ecular column along [010]. The columns are held together by O—H⋯O and N—H⋯N hydrogen bonds. An intra­molecular N—H⋯O inter­action also occurs.

## Related literature

For the structures of metal (*M*) complexes with the 3-amino­pyrazine-2-carboxyl­ate ligand, see: Leciejewicz *et al.* (1997 ▶ [*M* = Ca(II)], 1998 ▶ [*M* = Sr(II)]); Ptasiewicz-Bąk & Leciejewicz (1997 ▶ [*M* = Mg(II)], 1999 ▶ [*M* = Ni(II)]); Tayebee *et al.* (2008 ▶) [*M* = Na(I)]. For the structure of an Li(I) complex with pyrazine-2,3-dicarboxyl­ate and aqua ligands, see: Tombul *et al.* (2008 ▶). 
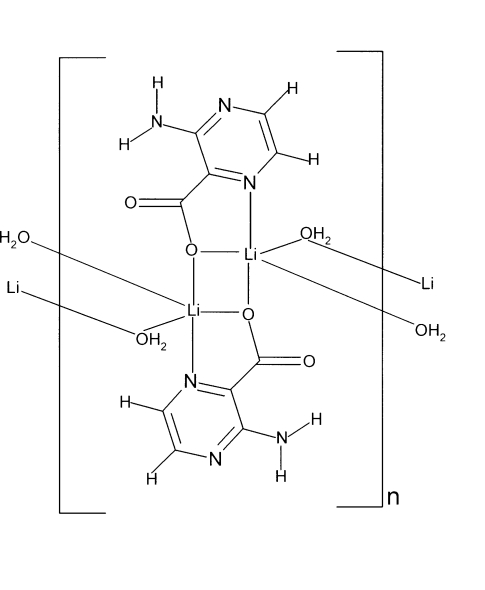


## Experimental

### 

#### Crystal data

[Li(C_5_H_4_N_3_O_2_)(H_2_O)]
*M* *_r_* = 163.07
Monoclinic, 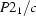

*a* = 14.279 (3) Å
*b* = 3.6000 (7) Å
*c* = 13.300 (3) Å
β = 106.43 (3)°
*V* = 655.7 (2) Å^3^
*Z* = 4
Mo *K*α radiation
μ = 0.13 mm^−1^
*T* = 293 K
0.26 × 0.21 × 0.04 mm

#### Data collection

Kuma KM-4 four-circle diffractometer
Absorption correction: analytical (*CrysAlis RED*; Oxford Diffraction, 2006 ▶) *T*_min_ = 0.980, *T*_max_ = 0.994
1997 measured reflections
1913 independent reflections
1297 reflections with *I* > 2σ(*I*)
*R*_int_ = 0.017
3 standard reflections every 200 reflections  intensity decay: 7.3%

#### Refinement

*R*[*F* ^2^ > 2σ(*F* ^2^)] = 0.049
*wR*(*F* ^2^) = 0.147
*S* = 1.04
1913 reflections
115 parameters
3 restraints
H atoms treated by a mixture of independent and constrained refinement
Δρ_max_ = 0.51 e Å^−3^
Δρ_min_ = −0.39 e Å^−3^

### 

Data collection: *KM-4 Software* (Kuma, 1996 ▶); cell refinement: *KM-4 Software*; data reduction: *DATAPROC* (Kuma, 2001 ▶); program(s) used to solve structure: *SHELXS97* (Sheldrick, 2008 ▶); program(s) used to refine structure: *SHELXL97* (Sheldrick, 2008 ▶); molecular graphics: *SHELXTL* (Sheldrick, 2008 ▶); software used to prepare material for publication: *SHELXTL*.

## Supplementary Material

Crystal structure: contains datablocks I, global. DOI: 10.1107/S1600536810020647/hy2312sup1.cif

Structure factors: contains datablocks I. DOI: 10.1107/S1600536810020647/hy2312Isup2.hkl

Additional supplementary materials:  crystallographic information; 3D view; checkCIF report

## Figures and Tables

**Table 1:** Selected bond lengths (Å)

Li1—N1	2.118 (3)
Li1—O1	1.999 (3)
Li1—O1^i^	1.995 (3)
Li1—O3	2.065 (3)
Li1—O3^ii^	2.201 (3)

Symmetry codes: (i) 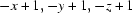; (ii) 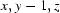.

**Table 2:** Hydrogen-bond geometry (Å, °)

*D*—H⋯*A*	*D*—H	H⋯*A*	*D*⋯*A*	*D*—H⋯*A*
O3—H31⋯O2^iii^	0.88 (1)	1.83 (1)	2.7028 (16)	175 (2)
O3—H32⋯O1^iv^	0.84 (2)	2.54 (2)	2.9083 (17)	108 (2)
N3—H1⋯O2	0.86	2.08	2.7229 (17)	131
N3—H2⋯N2^v^	0.86	2.30	3.1278 (19)	162

Symmetry codes: (iii) 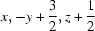; (iv) 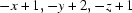; (v) 
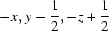
.

**Fig. 1.**
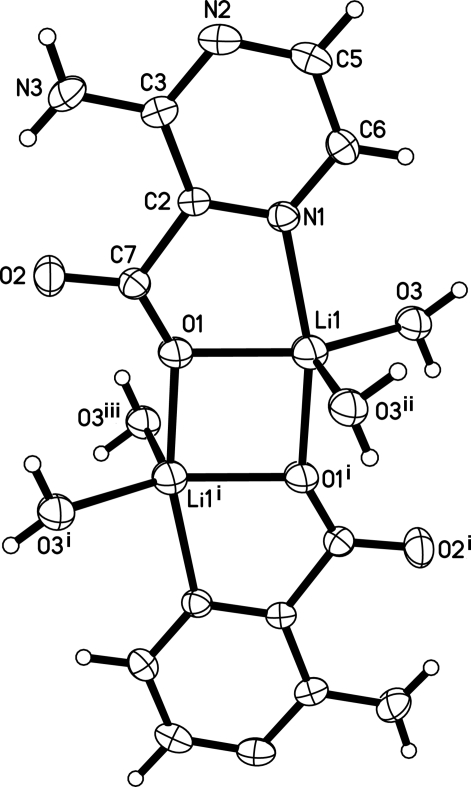
The dinuclear unit of the title compound. Displacement ellipsoids are drawn at the 50% probability level. [Symmetry codes: (i) 1-x, 1-y, 1-z; (ii) x, -1+y, z; (iii) 1-x, 2-y, 1-z.]

**Fig. 2.**
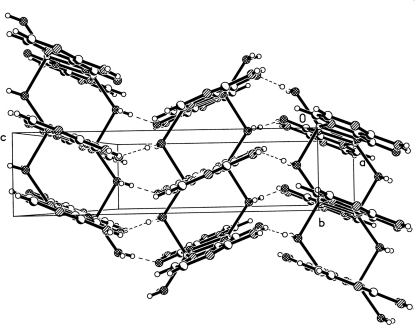
Packing diagram of the title compound.

## supplementary crystallographic information

### Comment

Structural studies of divalent metal ion complexes with 3-aminopyrazine-2-carboxylate ligand have shown that the structures of Mg(II) and Ni(II) complexes consist of M*L*_2_(H_2_O)_2_ monomers. In the Mg(II) comoplex, the ligand adopts a *cis* configuration (Ptasiewicz-Bąk & Leciejewicz, 1997), while in the Ni(II) complex, a *trans* configuration (Ptasiewicz-Bąk & Leciejewicz, 1999). Catenated polymeric molecular patterns have been reported in the structures of a Ca(II) complex (Leciejewicz *et al.*, 1997) and a Sr(II) complex (Leciejewicz *et al.*, 1998), in which metal ions are bridged by ligand carboxylate groups acting as bidentate. On the other hand, the structure of a Na(I) complex with the title ligand (Tayebee *et al.*,2008) is three-dimensional polymeric with Na(I) ions linked by an extended bridging system formed mainly by coordinated water O atoms.

The title compound is composed of centrosymmetric dinuclear units, in which each of the two Li^I^ ions is cheletated by a ligand *via* an N,O-bonding group. Its O atom acts as bidentate and bridges the other Li^I^ ion (Fig. 1). The dinuclear unit is nearly planar with r.m.s. of 0.0125 (2) Å. The Li^I^ ion is also coordinated by two water O atoms, which bridge the dinuclear units into molecular columns along two bridging pathways propagating in the *b*-axis direction (Fig. 2). The coordination geometry of the Li^I^ ion is trigonal bipyramidal, with the equatorial plane formed by O1, O3, O3^ii^ and with N1 and O1^i^ at the axial positions [symmetry codes: (i) 1-x, 1-y, 1-z; (ii) x, y-1, z]. The Li—O and Li—N bond distances (Table 1) and bond angles are typical for Li(I) complexes with carboxylate ligands (see, for example, Tombul *et al.*, 2008). The columns are linked by a network of hydrogen bonds, in which water O atoms are donors and the non-bonded carboxylate O atoms in adjacent columns act as acceptors. A weak hydrogen bond links an amino N atom with a hetero-ring N atom in the adjacent column. An intramolecular hydrogen bond which operates between the amino N3 atom and the non-bonding carboxylate O2 atom is also observed (Table 2).

### Experimental

The title compound was synthesized by reacting 50 ml of boiling aqueous solutions, one containing 1 mmol of 3-aminopyrazine-2-carboxylic acid (Aldrich), the other containing 1 mmol of lithium hydroxide (Aldrich). The mixture was boiled under reflux for 3 h and after cooling to room temperature, filtered and left to crystallize. A few days later, colourless single crystals in the form of flat needles were found after evaporation to dryness. They were extracted, washed with cold ethanol and dried in air. A crystal used for X-ray data collection was cut to adopt the shape of a flat plate.

### Refinement

Water H atoms were found from difference Fourier maps and their coordinates were refined with *U*_iso_(H) = 1.2*U*_eq_(O). H atoms attached to C and N atoms were positioned geometrically and refined as riding, with C—H = 0.93 and N—H = 0.86 Å and *U*_iso_(H) = 1.2*U*_eq_(C,N).

### Crystal data

**Table:**

[Li(C_5_H_4_N_3_O_2_)(H_2_O)]	*F*(000) = 336
*M**_r_* = 163.07	*D*_x_ = 1.652 Mg m^−^^3^
Monoclinic, *P*2_1_/*c*	Mo *K*α radiation, λ = 0.71073 Å
Hall symbol: -P 2ybc	Cell parameters from 25 reflections
*a* = 14.279 (3) Å	θ = 6–15°
*b* = 3.6000 (7) Å	µ = 0.13 mm^−^^1^
*c* = 13.300 (3) Å	*T* = 293 K
β = 106.43 (3)°	Plate, colourless
*V* = 655.7 (2) Å^3^	0.26 × 0.21 × 0.04 mm
*Z* = 4	

### Data collection

**Table:**

Kuma KM-4 four-circle diffractometer	1297 reflections with *I* > 2σ(*I*)
Radiation source: fine-focus sealed tube	*R*_int_ = 0.017
graphite	θ_max_ = 30.1°, θ_min_ = 1.5°
profile data from ω–2θ scans	*h* = −19→19
Absorption correction: analytical (*CrysAlis RED*; Oxford Diffraction, 2006)	*k* = −5→0
*T*_min_ = 0.980, *T*_max_ = 0.994	*l* = 0→18
1997 measured reflections	3 standard reflections every 200 reflections
1913 independent reflections	intensity decay: 7.3%

### Refinement

**Table:**

Refinement on *F*^2^	Primary atom site location: structure-invariant direct methods
Least-squares matrix: full	Secondary atom site location: difference Fourier map
*R*[*F*^2^ > 2σ(*F*^2^)] = 0.049	Hydrogen site location: inferred from neighbouring sites
*wR*(*F*^2^) = 0.147	H atoms treated by a mixture of independent and constrained refinement
*S* = 1.04	*w* = 1/[σ^2^(*F*_o_^2^) + (0.1051*P*)^2^ + 0.022*P*] where *P* = (*F*_o_^2^ + 2*F*_c_^2^)/3
1913 reflections	(Δ/σ)_max_ < 0.001
115 parameters	Δρ_max_ = 0.51 e Å^−^^3^
3 restraints	Δρ_min_ = −0.39 e Å^−^^3^

### Fractional atomic coordinates and isotropic or equivalent isotropic displacement parameters (Å^2^)

**Table:**

	*x*	*y*	*z*	*U*_iso_*/*U*_eq_	
C2	0.25443 (8)	0.4711 (3)	0.39664 (9)	0.0184 (3)	
O2	0.32090 (8)	0.2294 (4)	0.26435 (8)	0.0336 (3)	
N1	0.27984 (8)	0.6065 (3)	0.49343 (8)	0.0216 (3)	
N2	0.08397 (8)	0.5364 (4)	0.38343 (10)	0.0285 (3)	
N3	0.12597 (9)	0.2931 (4)	0.24154 (10)	0.0320 (3)	
H2	0.0649	0.2745	0.2090	0.038*	
H1	0.1690	0.2235	0.2115	0.038*	
O1	0.42229 (7)	0.4275 (4)	0.41421 (8)	0.0352 (3)	
C7	0.33887 (9)	0.3657 (4)	0.35429 (10)	0.0218 (3)	
C3	0.15415 (9)	0.4307 (4)	0.33936 (10)	0.0217 (3)	
C6	0.20958 (10)	0.7070 (4)	0.53653 (11)	0.0254 (3)	
H6	0.2262	0.8010	0.6044	0.030*	
C5	0.11268 (10)	0.6720 (4)	0.48077 (12)	0.0284 (3)	
H5	0.0653	0.7459	0.5123	0.034*	
Li1	0.43334 (19)	0.6091 (9)	0.5591 (2)	0.0370 (6)	
O3	0.44058 (8)	1.0866 (3)	0.64699 (9)	0.0338 (3)	
H32	0.4986 (11)	1.117 (6)	0.6825 (15)	0.041*	
H31	0.4047 (13)	1.146 (6)	0.6882 (14)	0.041*	

### Atomic displacement parameters (Å^2^)

**Table:**

	*U*^11^	*U*^22^	*U*^33^	*U*^12^	*U*^13^	*U*^23^
C2	0.0170 (5)	0.0153 (5)	0.0223 (6)	−0.0007 (4)	0.0048 (4)	0.0018 (4)
O2	0.0301 (5)	0.0450 (7)	0.0277 (5)	−0.0089 (5)	0.0114 (4)	−0.0120 (5)
N1	0.0209 (5)	0.0192 (5)	0.0242 (5)	0.0012 (4)	0.0057 (4)	−0.0006 (4)
N2	0.0194 (5)	0.0264 (6)	0.0390 (6)	0.0012 (4)	0.0071 (4)	0.0025 (5)
N3	0.0227 (5)	0.0401 (7)	0.0290 (6)	−0.0056 (5)	0.0002 (4)	−0.0052 (5)
O1	0.0183 (5)	0.0522 (7)	0.0338 (5)	0.0004 (5)	0.0052 (4)	−0.0142 (5)
C7	0.0202 (6)	0.0209 (6)	0.0242 (5)	−0.0021 (4)	0.0064 (4)	−0.0015 (5)
C3	0.0199 (5)	0.0171 (5)	0.0262 (6)	−0.0019 (4)	0.0033 (4)	0.0034 (5)
C6	0.0282 (6)	0.0225 (7)	0.0273 (6)	0.0020 (5)	0.0108 (5)	−0.0020 (5)
C5	0.0238 (6)	0.0237 (7)	0.0408 (8)	0.0031 (5)	0.0140 (5)	0.0006 (6)
Li1	0.0256 (12)	0.0484 (17)	0.0355 (13)	0.0016 (11)	0.0060 (10)	−0.0120 (12)
O3	0.0256 (5)	0.0411 (7)	0.0352 (6)	−0.0012 (5)	0.0092 (4)	−0.0069 (5)

### Geometric parameters (Å, °)

**Table:**

C2—N1	1.3274 (16)	C6—C5	1.378 (2)
C2—C3	1.4263 (17)	C6—H6	0.9300
C2—C7	1.5164 (17)	C5—H5	0.9300
O2—C7	1.2505 (17)	Li1—N1	2.118 (3)
N1—C6	1.3385 (17)	Li1—O1	1.999 (3)
N2—C5	1.335 (2)	Li1—O1^i^	1.995 (3)
N2—C3	1.3510 (18)	Li1—O3	2.065 (3)
N3—C3	1.3431 (18)	Li1—O3^ii^	2.201 (3)
N3—H2	0.8600	Li1—Li1^i^	2.900 (5)
N3—H1	0.8600	O3—H32	0.837 (15)
O1—C7	1.2515 (17)	O3—H31	0.875 (14)
			
N1—C2—C3	120.87 (11)	N2—C5—H5	118.6
N1—C2—C7	115.10 (11)	C6—C5—H5	118.6
C3—C2—C7	124.03 (11)	O1^i^—Li1—O1	86.88 (11)
C2—N1—C6	118.83 (11)	O1^i^—Li1—O3	94.05 (12)
C2—N1—Li1	111.64 (11)	O1—Li1—O3	142.73 (18)
C6—N1—Li1	129.48 (11)	O1^i^—Li1—N1	165.94 (15)
C5—N2—C3	117.50 (11)	O1—Li1—N1	79.08 (10)
C3—N3—H2	120.0	O3—Li1—N1	96.74 (12)
C3—N3—H1	120.0	O1^i^—Li1—O3^ii^	87.61 (12)
H2—N3—H1	120.0	O1—Li1—O3^ii^	102.21 (14)
C7—O1—Li1^i^	148.32 (12)	O3—Li1—O3^ii^	115.07 (14)
C7—O1—Li1	118.26 (11)	N1—Li1—O3^ii^	95.90 (13)
Li1^i^—O1—Li1	93.13 (11)	O1^i^—Li1—Li1^i^	43.49 (8)
O2—C7—O1	125.43 (12)	O1—Li1—Li1^i^	43.38 (8)
O2—C7—C2	118.94 (12)	O3—Li1—Li1^i^	126.66 (18)
O1—C7—C2	115.63 (11)	N1—Li1—Li1^i^	122.46 (16)
N3—C3—N2	117.93 (12)	O3^ii^—Li1—Li1^i^	96.72 (16)
N3—C3—C2	122.37 (12)	Li1—O3—Li1^iii^	115.07 (14)
N2—C3—C2	119.69 (12)	Li1—O3—H32	108.2 (15)
N1—C6—C5	120.32 (12)	Li1^iii^—O3—H32	94.4 (16)
N1—C6—H6	119.8	Li1—O3—H31	127.7 (15)
C5—C6—H6	119.8	Li1^iii^—O3—H31	100.2 (15)
N2—C5—C6	122.78 (13)	H32—O3—H31	106.0 (15)

Symmetry codes: (i) −*x*+1, −*y*+1, −*z*+1; (ii) *x*, *y*−1, *z*; (iii) *x*, *y*+1, *z*.

### Hydrogen-bond geometry (Å, °)

**Table:**

*D*—H···*A*	*D*—H	H···*A*	*D*···*A*	*D*—H···*A*
O3—H31···O2^iv^	0.88 (1)	1.83 (1)	2.7028 (16)	175 (2)
O3—H32···O1^v^	0.84 (2)	2.54 (2)	2.9083 (17)	108 (2)
N3—H1···O2	0.86	2.08	2.7229 (17)	131
N3—H2···N2^vi^	0.86	2.30	3.1278 (19)	162

Symmetry codes: (iv) *x*, −*y*+3/2, *z*+1/2; (v) −*x*+1, −*y*+2, −*z*+1; (vi) −*x*, *y*−1/2, −*z*+1/2.
